# Pain and anesthesia — on fetal nervous system and brain development

**DOI:** 10.3389/fphar.2025.1710759

**Published:** 2025-10-20

**Authors:** Mengjie Gao, Ganzhu Bao, Shuancheng Bai

**Affiliations:** 1 Affiliated Baotou Clinical College of Inner Mongolia Medical University, Baotou, China; 2 Department of Anesthesiology, Baotou Central Hospital, Baotou, China

**Keywords:** anethesia, nerve, brain, fetal, management of anesthesia during pregnancy

## Abstract

Does using narcotics for labor pain relief or undergoing general anesthesia during pregnancy affect fetal neurodevelopment? This is a critical concern for expectant mothers, families, and clinicians alike. While surgical interventions are typically performed during the second trimester—a critical phase for fetal central nervous system development—current research indicates that general anesthesia administered during this period may adversely impact the fetal CNS. How should we balance these risks? This review examines the latest studies on anesthesia’s effects on fetal neurodevelopment, exploring relevant mechanisms and characteristics. It aims to provide a comprehensive understanding and propose protective measures to ensure healthy fetal development while maintaining anesthesia safety throughout the entire pregnancy.

## 1 Introduction

General anesthesia, a widely used modern medical technique, works by suppressing central nervous system functions to induce unconsciousness, painlessness, and muscle relaxation. Its effects on the nervous system involve complex, multi-layered processes. The potential impact of general anesthesia during pregnancy on developing fetal nerves and brains has long been a subject of medical and public concern. While most current research focuses on newborns undergoing such procedures, many pregnant women in real-world practice require non-pregnancy-related general anesthesia for procedures including trauma surgeries, acute surgical conditions like appendicitis and cholecystitis, as well as fetal interventions. This review examines studies investigating whether general anesthesia during non-pregnancy surgeries affects fetal central nervous system development, aiming to provide a comprehensive understanding of this complex issue and ensure the health of both mother and fetus.

### 1.1 The mechanism of general anesthesia on the effect of nervous system development

The debate regarding the specific effects of general anesthesia on developing brains has persisted for over 3 decades. Current consensus indicates that prolonged, repeated, and high-dose exposure to anesthetics correlates with higher incidence of behavioral and executive function deficits, while single exposure shows relatively minor long-term neurofunctional impacts (Lu et al., 2026). A systematic review by Tom Bleeser and colleagues demonstrated that anesthesia exposure primarily affects learning and memory during brain development, with secondary effects observed in four biomarkers of neuronal damage (including apoptosis, synaptogenesis, neuronal density, and proliferation) (Bleeser et al., 2021).

Regarding learning and memory development: Song et al. demonstrated that maternal exposure to 2.5% sevoflurane during the first trimester of pregnancy causes fetal frontal nerve precursor cells to enter cell cycle arrest, reduces neuronal output from mentor neurons, and inhibits precursor cell replication, ultimately leading to learning deficits in offspring (Song et al., 2017). Furthermore, studies indicate that repeated maternal sevoflurane exposure suppresses Pax6 expression, thereby downregulating the cell cycle factor Ccnd1 and impairing neural development in fetal brains, resulting in cognitive dysfunction in offspring (Fang et al., 2017). Beyond rodent studies, single anesthetic exposure using isoflurane in gestational sheep models showed no significant neuronal apoptosis, but repeated anesthetic administration resulted in observable neuronal damage (Olutoye et al., 2016). These findings collectively reveal that prenatal anesthesia exposure, whether in terms of duration or frequency, exerts measurable impacts on offspring’s learning abilities, memory retention, and even cognitive functions.

Secondly, regarding the impact on neuronal injury markers: Neonatal anesthesia is associated with neuronal damage, such as increased apoptosis, impaired dendritic and axonal branching, and altered neural generation (Satomoto et al., 2009; Lee et al., 2014; Mintz et al., 2013). During mid-pregnancy, the nervous system primarily undergoes neural and glial production and neuronal migration before synapse formation. Neural and glial production refers to the proliferation and differentiation of neurons and glial cells from pluripotent neural stem (precursor) cells. This process begins in late embryonic development and continues through the second and third trimesters during pregnancy (Leibovitz et al., 2022). Additionally, neuronal migration plays a crucial role in normal cerebral cortex development. This process involves mitotically active subventricular zone neurons migrating toward the outer regions of the developing brain. Glial cells (astrocytes and oligodendrocytes) proliferate and differentiate during the first 3 months of pregnancy, while myelination of nerve fibers predominantly occurs postnatally (Govaert et al., 2020). Abnormal progression of embryonic and fetal central nervous system development may lead to specific congenital central nervous system abnormalities, many of which have potential genetic variations and are associated with developmental delays and intellectual disabilities (Leibovitz et al., 2022). Impaired neurotransmitter signaling and functional deficits in neural networks are core features of developmental disorders. Potential mechanisms include genetic and environmental factors. Experimental evidence indicates that early anesthesia exposure induces persistent morphological and functional changes in neural networks (Vutskits and Xie, 2016). GABAAR agonists and other anesthetics directly act on GABAAR in the central nervous system, inducing reversible loss of consciousness, amnesia, and anesthesia. Studies show that GABAAR agonist sedatives exhibit dual effects on developing brains: as total anesthetic doses increase, their effects transition from protective to neutral, ultimately becoming destructive (Lu et al., 2026). The low-dose protective effect we previously observed may represent a new research direction.

### 1.2 Characteristics of fetal nervous system development

The human brain is “the most complex object in the known universe, with billions of cells and trillions of connections that constitute a true marvel.” The formation of the human nervous system is a continuous, precise, and orderly process—— where neurons are precisely positioned to establish synaptic connections with other cells in specific locations (Sanes et al., 2006). The development of the nervous system during fetal development is crucial throughout life, particularly during the second trimester (around 3 months), as synaptic formation primarily occurs during the mid-to-late stages of human pregnancy. In rodents, however, synaptic formation begins postnatally (Dobbing and Sands, 1979). Prior to synaptic formation, the main components of neural development are neural generation and neuronal migration, activities that peak during the second trimester. During fetal brain development, neural stem cells proliferate at a rate of millions per minute or even per second, forming numerous neurons that merge with others and migrate to establish neural circuits (Avet-Rochex et al., 2014). During this critical period, neural development, neural generation, and neuronal migration are highly sensitive to environmental changes and drug effects. Taken together, these findings indicate that the regulation of neural generation is essential for functional nervous system formation, particularly the balance between cell proliferation, differentiation, and survival.

The third trimester of pregnancy is a critical period for the development of the prefrontal cortex (PFC), which plays a key role in the pathogenesis of advanced cognitive functions and various neurodevelopmental disorders (Huang and Vasung, 2014). Moreover, during late pregnancy and infancy, neuronal apoptosis is a natural physiological process—— affecting approximately 1% of neurons daily (Ista et al., 2013).

### 1.3 Characteristics of anesthesia during pregnancy

We must emphasize that anesthesia administration primarily targets a specific population——pregnant women. Their unique physiological and anatomical characteristics should not be overlooked. As the vital regulator of maternal-fetal health, the placenta serves as one of the most crucial organs sustaining fetal life throughout pregnancy (Arumugasaamy et al., 2020). Therefore, particular attention should be paid to how the placental barrier affects drug transport. Placental drug transfer depends on multiple factors: lipophilic drugs with high solubility allow rapid penetration but may become trapped in the placenta, while protein binding varies depending on drug-protein interactions (Upadya and Saneesh, 2016). Since most anesthetic drugs are lipophilic (Zheng et al., 2013), they easily cross the placenta through simple diffusion. Studies indicate that isopropylphenol undergoes rapid absorption and tissue distribution during intravenous anesthesia in pregnancy, leading to accelerated maternal-fetal balance and immediate reduction in fetal blood concentration postpartum. Intravenous continuous infusion may affect fetal tissue metabolism or sustained absorption (SciELO, 2025). Similarly, ketamine—a highly lipophilic drug—shows higher fetal blood concentrations than maternal plasma (Upadya and Saneesh, 2016) (with a F/M ratio of 1.26 observed within 2 min after intravenous bolus). In contrast, etomidate demonstrates a steep decline in plasma concentration following single-dose administration during pregnancy (ranging from 1,242.0 ng/mL at 5 min post-injection to undetectable levels within 2 h), resulting in a umbilical-cord-to-maternal-blood ratio of 1:24 (Esener et al., 1992), indicating that etomidate can be used for anesthesia during pregnancy. Finally, the commonly used muscle relaxants (depolarizing agents: e.g., succinylcholine; non-depolarizing agents: e.g., rocurbamine) are mostly water-soluble drugs with minimal impact on the fetus (Kampe et al., 2003).

### 1.4 Effects of anesthesia exposure during pregnancy on fetal nervous system development

Numerous studies have demonstrated that general anesthesia may adversely affect the developing brain. However, the complexity of research on fetal exposure to anesthesia has led to ongoing debates in this field. While randomized controlled trials are recognized as the gold standard, ethical and technical constraints currently limit our ability to study anesthetic effects on fetuses using relevant animal models (Li et al., 2021). Consequently, most current research on intraoperative anesthesia during pregnancy relies on various animal experiments. Whether fetal anesthesia exposure impacts neural development requires further investigation in future studies (Andropoulos, 2018).

In addition to the effects of the placental barrier on drugs mentioned in the previous section, we need to consider the dosage and duration of use of anesthetic drugs, at which stage of pregnancy the drugs are used, and the effects of these factors.

Regarding the stages of pregnancy,It is generally considered safe to perform surgery during the mid-to-late stages of pregnancy. However, studies indicate that this period coincides with a critical phase of accelerated neural development and neuronal migration in fetal brains (IJFM R, 2023). Exposure to anesthetics during these trimesters may lead to neuroinflammation, apoptosis, synaptic loss, and cognitive impairments in offspring. These findings have raised concerns about the safety of anesthesia use during mid-pregnancy. Van der Veeken et al. induced general anesthesia in rabbits at 28 days of gestation (equivalent to the second to third trimester in humans) using isopropylphenol, followed by a 2-h laparotomy surgery. Their results showed delayed neurofunctional development and brain morphological changes in the offspring (Van der Veeken et al., 2019). Additionally, research teams discovered that general anesthesia during the second to third trimester causes apoptosis and affects neural crest cell (NSC) proliferation, potentially leading to long-term learning and behavioral abnormalities in offspring (Wang et al., 2018a). Furthermore, studies in late-pregnancy sheep models revealed that prolonged systemic isoflurane anesthesia significantly reduces uterine blood flow, triggering fetal peripheral vasoconstriction and potentially causing severe acidosis (Shaw et al., 2020). De Tina et al. found that fetal brains in late-pregnancy rodents and non-primate species are particularly vulnerable to the effects of inhalation and intravenous anesthetics, with this vulnerability becoming more pronounced during prolonged administration. Moreover, fetal brains in the third trimester may be more susceptible to adverse effects from inhalation anesthetics (Li et al., 2021). Studies have shown that exposure to sevoflurane during pregnancy may cause neurotoxic effects on fetal brain development (Zuo et al., 2021), though the specific mechanisms and target sites remain unclear. This highlights a critical awareness: even relatively safe surgical procedures may not ensure fetal nervous system safety. The second trimester is a critical period for neural formation and migration, during which the fetus’s nervous system remains highly vulnerable to environmental influences. Even subtle changes in this phase could lead to long-term neurological abnormalities.

Secondly, regarding the duration of anesthetic exposure, most current studies confirm that anesthesia exposure time is a critical factor affecting fetal neurodevelopment. The U.S. Food and Drug Administration (FDA) has issued warnings that repeated or prolonged (>3 h) anesthesia exposure during pregnancy may impair fetal brain development (Olutoye, 2018). A meta-analysis demonstrated that repeated or extended (>3 h, equivalent to 40–47 h of human anesthesia exposure) whole-body anesthesia or exposure exceeding 1 minimum alveolar concentration (MAC) during pregnancy in pregnant mice caused neuronal damage and neurobehavioral disorders (Bleeser et al., 2021). However, single brief (≤3 h) exposure to volatile anesthetics at ≤1 MAC levels did not lead to significant neurodevelopmental impairments (Bleeser et al., 2021). Additionally, research findings indicate that while a single sevoflurane exposure does not affect offspring rats’ learning and memory abilities, repeated sevoflurane exposure results in impaired cognitive functions (Wu et al., 2018). This suggests that the frequency of anesthesia exposure is another crucial risk factor.

Finally, regarding the dosage of anesthetic drugs, there is currently no established safe range or usage guidelines for their application during pregnancy. Studies have shown that mid-pregnancy exposure to sevoflurane can induce apoptosis in neural stem cells (NSCs) and impair postnatal learning and memory functions in a dose-dependent manner (Wang et al., 2018a). Additionally, mid-pregnancy administration of 3.5% sevoflurane inhibits fetal NSC proliferation (Wang et al., 2018a). High concentrations of anesthetics are known to adversely affect hemodynamics in both fetuses and mothers (Okutomi et al., 2009). Furthermore, volatile anesthetics like sevoflurane exacerbate neuronal cell death during development (Liu et al., 2015). Notably, a study demonstrated that high-dose dexmedetomidine exposure for 12 h during mid-pregnancy did not affect neurocytotoxicity in the cerebral frontal cortex of fetal dogs and monkeys (Koo et al., 2014). By combining the characteristics of inhalation and intravenous anesthetics, we can implement combined anesthesia regimens to minimize adverse effects (Lépiz et al., 2017). With no definitive safety thresholds defined for pregnancy-related anesthetic dosing, future research should prioritize this critical area to ensure clinical anesthesia safety and maternal-fetal wellbeing.

Beyond the aforementioned findings, experimental evidence demonstrates that general anesthesia administered during pregnancy causes fetal neuronal damage in animal studies regardless of species or gestational stage. While the neurotoxic effects induced by anesthesia during pregnancy are consistent with laboratory conditions, their impact on neural development diminishes significantly in studies simulating clinical scenarios using standard monitoring protocols (Bleeser et al., 2021). Therefore, translating laboratory data into complex human clinical environments is essential to enhance the relevance of experimental findings. Additionally, pregnancy-related conditions such as hypertension and anemia may contribute to fetal abnormalities or preterm delivery, with the exact role of anesthesia remaining unclear (Li et al., 2021). To date, no definitive reports exist regarding the consequences of general anesthesia on fetal neurodevelopment. Consequently, future research must comprehensively investigate the potential neurotoxicity and long-term effects of fetal anesthesia exposure, aiming to minimize its safety risks and health impacts on both pregnant women and developing fetuses.

### 1.5 Prognosis and clinical consensus of anesthesia exposure during pregnancy

A study demonstrated measurable effects on neonatal neurological function and brain morphology during 2-h maternal general anesthesia and surgery in rabbits. Although the motor nerve development of young rabbits progressed slowly, these effects became nearly undetectable by week 7 (Van der Veeken et al., 2019). Another research revealed that maternal exposure to sevoflurane during the first trimester could lead to abnormal neuronal differentiation in fetal brains. However, subsequent recovery observed in later stages suggests sevoflurane exposure might not have lasting impacts on fetal brain development (Lee et al., 2022). The long-term effects of prenatal anesthesia on fetuses remain uncertain. Current research indicates that such interventions pose certain risks to both mothers and fetuses.

First, elective procedures should be avoided during pregnancy whenever possible. When appropriate, regional anesthesia should be the primary choice for maternal care (Flood and Flood, 2011). Second, general anesthesia—particularly inhalation anesthesia—remains a common method for emergency non-obstetric surgeries during the second trimester. Currently, the U.S. Food and Drug Administration (FDA) has not issued recommended concentrations or guidelines to prevent fetal neurodevelopmental damage. Therefore, when general anesthesia is necessary, we should minimize its duration and use lower concentrations of anesthetics to reduce exposure for both mother and fetus. Moving forward, our research should focus on developing safer anesthesia techniques, strategies, and medications. We must prioritize the safety of both mothers and fetuses while minimizing fetal trauma and interventions.

### 1.6 Summary and outlook

The management of anesthesia during non-obstetric procedures in pregnant women constitutes a complex, multidisciplinary field requiring comprehensive consideration of maternal and fetal safety. Our primary objectives are to safeguard the healthy development of the fetus, prevent both immediate and long-term adverse effects, while implementing flexible adjustments in specific clinical scenarios. During surgical procedures, we must closely monitor cardiovascular stability, optimize uterine perfusion, ensure adequate uterine relaxation, maintain sufficient anesthesia depth, minimize fetal myocardial inhibition, and appropriately manage fetal stress responses (Sviggum and Kodali, 2013). Current intraoperative fetal monitoring methods include Doppler ultrasound, uterine contraction monitoring, and advanced techniques such as the Power-MF fetal electrocardiographic monitoring system (Jaeger et al., 2024), along with other sophisticated, safe, and accurate comprehensive monitoring approaches. Additionally, selecting anesthetics with neuroprotective effects is crucial. For instance, growing evidence indicates that diltiazem demonstrates neuroprotective efficacy in pediatric and adult populations (Tsivitis et al., 2023). Moreover, close collaboration among multidisciplinary medical teams is essential. This synergy ensures comprehensive care for expectant mothers, enhances surgical efficiency, reduces operation time, and minimizes anesthesia exposure–all critical factors for optimal outcomes. However, translating animal research findings into human studies remains challenging, making further investigation into the potential neuroprotective effects of dextromethorphan anesthetic a promising research frontier. As medical science advances, future efforts should focus on animal experiments to deepen our understanding of this complex subject, develop safer anesthesia techniques, strengthen the implementation of gold-standard randomized controlled trials at ——centers, thereby obtaining authoritative conclusions and providing clinical guidelines.

Modern anesthesia techniques have significantly reduced risks. For epidural anesthesia commonly used during pregnancy, we employ real-time ultrasound visualization of spinal structures to achieve precise puncture and anesthesia, enhancing both success rates and safety. In emergency general anesthesia procedures, we implement combined medication regimens to minimize adverse effects on both mother and baby. For challenging airways and special maternal cases, we utilize multimodal labor analgesia combined with neurotonic general anesthesia to reduce drug consumption while promoting rapid recovery. Additionally, we apply intelligent anesthesia depth monitoring to dynamically adjust anesthetic infusion rates, enabling personalized precision anesthesia. However, the administration of anesthesia and surgical procedures during pregnancy still requires careful risk-benefit assessment. Future research will further elucidate the interaction mechanisms between anesthetic drugs and fetal neurological systems, while continuously refining clinical practices to safeguard the health of both mother and fetus.
